# System quality, information quality, satisfaction and acceptance of online learning platform among college students in the context of online learning and blended learning

**DOI:** 10.3389/fpsyg.2022.1054691

**Published:** 2022-12-16

**Authors:** Xiaoxia Li, Wanxia Zhu

**Affiliations:** College of Educational Science and Technology, Northwest Minzu University, Lanzhou, China

**Keywords:** online learning platform, online learning, blended learning, system quality, information quality, USATA

## Abstract

The paper was based on the User Satisfaction and Technology Acceptance Integration Theory (USATA). The authors analyzed the factors that affect college students’ acceptance and satisfaction of online learning platform, as well as the differences in the relationship between various factors in blended learning scenario and online learning scenario. The results showed that the quality of online learning platform and information quality affect user satisfaction, and satisfaction affects usefulness and ease of use, and then affect attitude and intention. The comparison between the two groups showed that there were significant differences in the impact of information quality on information satisfaction and the impact of perceived usefulness on usage intention. In the online learning scenario, the endogenous latent variables of the model had higher explanatory power, which indicates that learners are more dependent on the quality and relevant characteristics of the learning platform in the online learning scenario.

## 1 Introduction

Since 2019, the novel coronavirus (COVID-19) disease has been pandemic in the world. The whole world has taken many measures to prevent the spread of COVID-19. The relevant measures have had a great impact on our social, economic life, health, work, and learning (Clark et al., 2021). The United Nations Education, Scientific and Cultural Organization (UNESCO) pointed out that the COVID-19 has affected the world’s education system (UNESCO, 2020). In order to reduce the impact on students’ learning during school closure, many countries used information technology and online learning platforms or tools to carry out massive online teaching and learning (Varalakshmi and Arunachalam, 2020). The Chinese Ministry of Education has launched the initiative of “Disrupted Classes, Undisrupted Learning” to provide students with flexible online learning (Huang et al., 2020).

During the COVID-19, online learning became an alternative to face-to-face learning in schools. Online learning platform had become an important way and tool for learners to learn in the completely home-based online learning environment. This had led to a large number of studies focusing on learners’ acceptance and satisfaction with the online learning platform in the online learning environment. For example, some researchers used the extended Technology Acceptance Model (TAM) or the Unified Theory of Acceptance and Use of Technology (UTAUT) to study learners’ acceptance of online learning or online learning platform during the COVID-19 (Aguilera-Hermida, 2020; Mailizar et al., 2021; Raza et al., 2021). During the period of COVID-19 and school closure, online learning platform is a key factor for the massive online learning. These studies showed that learners’ acceptance and satisfaction with online learning platforms will affect learners’ intention to use online learning platforms and online learning effect.

In the blended learning scenario during the non-COVID-19 period, learners also need to use the online learning platform to carry out online learning. Unlike online learning, blended learning includes not only online learning, but also face-to-face learning. Many studies had explored learners’ acceptance and satisfaction with online learning platforms in blended learning environments. For example, some researchers use the extended TAM model (Padilla-Meléndez et al., 2013; Al-Azawei et al., 2017), Expectation Confirmation model (Cheng, 2014), and the Unified Technology Acceptance and System Success model (Zhang et al., 2020) to investigate the intention of college students to use the blended learning system. Some researchers use similar models to investigate the acceptance and satisfaction of knowledge management systems in blended learning environments (Bervell and Umar, 2020; Ustun et al., 2021).

In conclusion, blended learning and online learning scenarios are widely used. The teaching scenarios have an important impact on students’ learning experience and learning quality (Lim and Morris, 2009; Gómez-Rey et al., 2016). In both learning scenarios, online learning platform is an important learning resource and tool. At present, most researches focus on the acceptance or satisfaction of a learning scenario, less on the combination of satisfaction and acceptance, and pay attention to the differences in different learning scenarios. This study attempts to analyze the college students’ acceptance, satisfaction and influence factors of online learning platform in the two scenarios of blended learning and online learning, and compare the differences in the relationship between various factors in the two scenarios. This study aims to provide theoretical basis for the development of online learning platform and the design of teaching resources in online learning platform.

## 2 Literature review

### 2.1 User satisfaction theory

Previous studies focusing on the success factors of information systems have found that system quality, information quality and user satisfaction are the key factors that affect the success of information systems (DeLone and McLean, 1992). In a specific context, the satisfaction is the sum of a person’s feelings toward various factors that affect the situation (Schwab and Cummings, 1970). User Satisfaction in this study is defined in the specific context of using information systems or platforms. Satisfaction is the sum of a person’s positive and negative reactions to a series of factors. User Satisfaction Model mainly lists some attributes of system and information design, which are used as factors affecting satisfaction (Wanous and Lawler, 1972).

Through the comprehensive analysis of the researches on the evaluation tools of user information system satisfaction, it is found that the common dimensions of information system user satisfaction measurement tools are system quality and information quality. System quality includes five sub dimensions, namely reliability, flexibility, integration, accessibility and timeliness. Information quality includes four sub dimensions, namely integrity, accuracy, format and currency (Bailey and Pearson, 1983). Reliability refers to the reliability or stability of system operation. Flexibility means that the system can meet the changing needs of users. Integration refers to the integration or compatibility of the system, allowing the integration of data from different sources. Accessibility refers to the ease of use of the system, which is convenient to access the system or extract information. Timeliness refers to the system’s timely response to user requests. Integrity means that the information provided by the system is necessary and comprehensive. Accuracy refers to the scientific and correct information provided by the system. Format refers to the standard and reasonable presentation of information provided by the system. Currency means that the information provided by the system is updated in time (Ives et al., 1983; Baroudi and Orlikowski, 1988). It can be seen that the user satisfaction model focuses on the characteristics of the system and information. User satisfaction is regarded as the attitude of users toward information systems, and it can also be regarded as an object-based attitude (Ajzen and Fishbein, 1980). User satisfaction is mainly measured by various object-based beliefs such as the quality of system technology and services, and the quality of information carried and transmitted by the system (Wixom and Todd, 2005). However, user satisfaction cannot predict users’ use of the system well, that is, users who are satisfied with the system and information do not necessarily use the system (Barki, 1994).

In this study, online learning platform is regarded as an information system. Therefore, based on the above literature review, the following hypotheses are put forward by the authors:

H1: Information Quality positively influences Information Satisfaction.

H2: System Quality positively influences System Satisfaction.

### 2.2 Technology acceptance theory

The commonly used model for investigating technology acceptance is the TAM proposed by David. The TAM model mainly includes the following variables and relationship of variables: (1) Whether users use the system depends on the user attitude toward the system. (2) User attitude toward the system will affect users’ intention to use it. (3) Attitude is mainly influenced by behavioral beliefs such as perceived usefulness and perceived ease of use. (4) Perceived ease of use will affect perceived usefulness (Davis, 1985). Perceived usefulness refers to the extent to which a person believes that using a specific system can improve job performance. Users think that if using a system can help improve job performance, they think it is a system with high perceived usefulness. Perceived ease of use refers to the degree to which a person thinks that using a particular system will save effort. Users think that if this system is easier to use, it will be more easily accepted by users (Davis, 1989). The TAM model is widely used in the research of understanding people’s attitude toward technology use, and is mainly used to predict the users’ use of information technology and tools. The TAM model only provides suggestions on how to improve users’ use by designing and perfecting the system (Taylor and Todd, 1995). Based on this model, the designer can generally receive user feedback on the ease of use and usefulness of the system or platform, but will not receive feedback on the characteristics of the system or platform itself, such as flexibility, integration, reliability, information integrity, etc.

Therefore, based on the above literature review, the following hypotheses are put forward by the authors:

H3: Perceived ease of use positively influences perceived usefulness.

H4: Perceived usefulness positively influences user attitude.

H5: Perceived usefulness positively influences usage intention.

H6: Perceived ease of use positively influences user attitude.

H7: User attitude positively influences usage intention.

### 2.3 User satisfaction and technology acceptance integration theory

Among the relevant TAM studies, some studies have focused on the key factors that affect ease of use and usefulness, such as gender, social impact and other factors (Gefen and Straub, 1997; Venkatesh and Morris, 2000). Venkatesh et al. (2003) empirically compared eight models in the field of information system technology acceptance, including rational behavior theory, technology acceptance model, motivation model, planned behavior theory, a model combining TAM and planned behavior theory, personal computer utilization model, innovation diffusion theory and social cognition theory, and developed the UTAUT. At the same time, based on the Technology Acceptance Model, they verified the external variables that affect the behavioral intention of digital libraries, such as individual differences and system characteristics. System characteristics mainly include relevance, terminology, and screen design. Individual differences and system characteristics have significant effects on perceived ease of use, thus affecting behavioral intention. System characteristics have significant effects on perceived usefulness, thus affecting behavioral intention. In particular, the relevance of system characteristics has the greatest effect on perceived usefulness (Hong et al., 2002).

According to the expectancy-value theory (Wigfield and Eccles, 2000), external variables will affect the user belief in performing a certain behavior, thus affecting the attitude of performing a certain behavior. Attitude will affect the intention to perform the behavior, and ultimately affect the behavior itself. Under certain circumstances, satisfaction is the feeling toward these external factors (Ajzen and Fishbein, 1980). External factors such as system characteristics will affect behavioral beliefs such as perceived ease of use or perceived usefulness (Hong et al., 2002). It can be seen that user satisfaction is the feeling toward external factors such as system characteristics and information characteristics. The beliefs of system quality and information quality will affect user attitude toward the system or information, so it will affect behavioral beliefs about using the system, such as perceived ease of use and usefulness. The behavior belief of using system directly affects the user attitude, and finally affects the behavior intention (Wixom and Todd, 2005).

The TAM can predict users’ intentions and behaviors in a specific context and time according to the specific behavior belief and attitude, but it cannot obtain feedback on the characteristics of the system itself. User Satisfaction Model can obtain the characteristics of the system and information, but it cannot predict user behavior well. The characteristics of information and system will affect their satisfaction. Satisfaction may affect their behavioral beliefs about the system or information, and then affect their behavioral attitude, thus affecting usage intention (Ajzen and Fishbein, 1980). Wixom and Todd (2005) proposed and verified the Theoretical Integration of User Satisfaction and Technology Acceptance (USATA), which can better integrate the advantages of User Satisfaction and Technology Acceptance Model, and build the relationship between the two models. Information quality and system quality represent object-based belief. Satisfaction with information and systems represents object-based attitude. Object-based attitude is external variable of behavioral belief such as perceived ease of use and perceived usefulness. Specifically, the higher the system satisfaction, the more users will feel that the system is easy to use. The higher the information satisfaction, the more users will feel that the application of this information is useful for their work. In the Technology Acceptance Model, perceived ease of use affects perceived usefulness. Consistent with this view, the model proposes that system satisfaction affects information satisfaction.

Therefore, based on the above literature review, the following hypotheses are put forward by the authors:

H8: Information satisfaction positively influences perceived usefulness.

H9: System satisfaction positively influences perceived ease of use.

H10: System satisfaction positively influences information satisfaction.

## 3 Materials and methods

### 3.1 Participants

Two groups (BL group and OL group) were selected from a public university in Lanzhou, China; they came from the same educational technology major and were taught by the same lecturer. Two groups studied the same course in different years. BL group participants studied this course in blended learning environment, who started their university studies in 2015 and 2016. OL group participants studied this course in online learning environment, who started their university studies in 2017 and 2018. The two groups of participants had the same professional learning experience and were familiar with the online learning platform they used. BL group had 143 students (43 male students and 100 female students). OL group had 134 students (34 male students and 100 female students). When they studied this course, they were in their junior year, ranging in age from 19 to 21.

### 3.2 Setting

This study was conducted in the form of quasi-experiment. Both groups took the same courses, had the same teachers, and used the same online learning platform. They were just different in the design of the learning environment.

The course was called “Design and Development of Multimedia Curriculum Resources,” which aims to enable students to master the design and development methods of different types of curriculum resources, so as to make curriculum resources suitable for future teaching. Teachers of this course had 11 years of teaching experience and was exploring new teaching methods. The online learning platform used in this course was Chaoxing Fanya Platform. It was an online learning platform developed by China Chaoxing company. Teachers could set up courses on this platform, add courseware, test questions, teaching videos and other course resources, and carried out online activities such as topic discussion, grouping tasks, assignments, and evaluation.

During the non-COVID-19 epidemic period, the author carried out blended learning method relying on the Chaoxing Fanya platform. Before the COVID-19, in October 2019, the author conducted a survey (called BL group) in order to find out the factors affecting user satisfaction and acceptance of the online learning platform in blended learning scenario.

Due to the COVID-19, the author’s university carried out four times of complete online teaching at home. The authors find that the importance of online learning platforms becomes more prominent when learning is completely online. In complete online learning, learners can only rely on online learning platform to obtain course knowledge, so the quality of online learning platform will affect students’ learning process. Therefore, in December 2021, the authors also conducted a survey (called OL group) on the user satisfaction and acceptance of the online learning platform for learners who used the same Chaoxing Fanya platform in online learning scenario.

### 3.3 Treatment

The BL group was taught before the COVID-19, using blended learning approach. When face-to-face teaching, the teacher mainly explained the key and difficult points of the curriculum theory and students’ difficult problems. In addition, in the course practice part, students practiced the development process of teaching resources. Before or after class, students could preview or review relevant resources of the course with the help of Chaoxing Fanya Platform; complete the after-school grouping tasks, participate in group theme discussion, participate in the outcome evaluation and other activities. Teachers conducted online guidance and evaluation on students’ grouping tasks.

The OL group was taught during the COVID-19, and adopted a complete online learning method. The author used the curriculum resources built by Chaoxing Fanya Platform, and carried out online learning with the help of Chaoxing classroom and live broadcast software. Before class, students learned relevant courseware and videos in the online platform, completed the test questions, and put forward learning questions in the discussion area. In class, Teachers used live broadcast software to answer students’ questions before class; students presented their learning achievements and exchanged comments. After class, students could watch the live broadcast course playback and course materials, review the course content, complete the homework, group tasks, and participate in online evaluation.

### 3.4 Instruments

The survey scale of this study mainly referred to the measurement scales of the Theoretical Integration of User Satisfaction and Technology Acceptance (Wixom and Todd, 2005). The questionnaire was divided into eight dimensions, namely information quality (3 survey items), system quality (3 survey items), information satisfaction (2 survey items), system satisfaction (2 survey items), user attitude (3 survey items), use intention (3 survey items), perceived ease of use (3 survey items) and perceived usefulness (3 survey items), totaling 22 items (see Appendix Table A1). Each item on the scale was measured on a seven-point Likert scale ranging from 1 (strongly disagree) to 7 (strongly agree). According to the theme of this study, the information system was defined as the Chaoxing Fanya platform, and some modifications were made according to the Chinese background, which is more convenient for Chinese students to understand. All scales in this study have implemented the back-translation procedure (Brislin, 1970).

### 3.5 Why PLS-SEM

In this study, Partial Least Squares Structural Equation Modeling (PLS-SEM) method was used for data analysis, and the analysis tool was SmartPLS 2.0. The PLS-SEM algorithm was selected in this study mainly because the PLS-SEM algorithm is very suitable for the study of small-scale samples (Subramani, 2004; Urbach and Ahlemann, 2010). Rules of thumb given by previous researchers suggested that the sample size in PLS-SEM model should be five times the largest number of independent variables (Falk and Miller, 1992), or equal to 10 times the number of independent variables in the most complex regression in the PLS path model (i.e., considering both measurement and structural models) (Hair et al., 2021). Some researchers believe that in PLS-SEM research, the appropriate sample size depends on many factors, such as the psychometric properties of the items, the effect size of the model, and the characteristics of the raw data (Chin and Newsted, 1999; Marcoulides and Saunders, 2006). Chin and Newsted (1999) suggested that the PLS-SEM algorithm can get accurate parameter estimates at sample size as low as 20. In this study, the sample sizes of BL group and OL group were 143 and 134 respectively, which is enough. The data analysis process mainly includes two steps. The first step is to analyze the measurement model to evaluate the reliability, internal consistency reliability, convergence validity and discrimination validity of the model. The second step is to analyze the structural model to evaluate the goodness-of-fit, coefficient of determination, path coefficient and group comparison results of the model (Hair et al., 1998).

## 4 Results

### 4.1 Measurement model

The evaluation of the measurement model is carried out through four aspects: item reliability, internal consistency reliability, convergence validity and discrimination validity.

#### 4.1.1 Item reliability

The item reliability was evaluated by the indicator loadings. The reliability of one structure is independent of other structures and calculated separately from the reliability of other structures. According to Chin’s suggestion, the factor loadings should be greater than 0.7 (Chin, 1998). As showed in Table 1, all indicator loadings in this study met the requirement. The ranges of item loadings in BL group and OL group were (0.799, 0.942) and (0.883, 0.959) respectively, which were greater than the recommended value. Overall, the item reliability in research models of the BL group and OL group was supported.

**TABLE 1:** Cronbach’s alpha, composite reliability, average variance extracted (AVE), factor loadings of the constructs and items in the research models of BL and OL.

	Cronbach’s alpha/Composite reliability/AVE		Factor loadings		M		SD	
	BL	OL	BL	OL	BL	OL	BL	OL
Information quality (INQU)	0.906/0.941/0.842	0.931/0.956/0.879						
INQU1			0.931	0.946	5.350	5.313	1.096	1.007
INQU2			0.924	0.937	5.448	5.306	1.053	1.013
INQU3			0.898	0.930	5.294	5.284	1.087	1.016
System quality (SYQU)	0.920/0.950/0.863	0.916/0.947/0.857						
SYQU1			0.937	0.931	5.273	5.231	1.127	0.957
SYQU2			0.942	0.917	5.357	5.336	1.141	0.925
SYQU3			0.907	0.929	5.357	5.336	0.989	0.934
Information satisfaction (INSA)	0.820/0.918/0.848	0.910/0.957/0.917						
INSA1			0.918	0.957	5.573	5.313	0.968	0.953
INSA2			0.923	0.959	5.336	5.336	1.041	0.973
System satisfaction (SYSA)	0.862/0.936/0.879	0.886/0.946/0.898						
SYSAT1			0.938	0.948	5.357	5.306	1.058	0.952
SYSAT2			0.937	0.947	5.343	5.328	1.015	0.899
Usefulness (USEF)	0.898/0.936/0.831	0.942/0.963/0.896						
USEF1			0.906	0.937	5.476	5.343	0.999	0.982
USEF2			0.899	0.944	5.392	5.396	1.081	1.026
USEF3			0.929	0.958	5.350	5.410	0.959	0.990
Ease of use (EAOU)	0.804/0.884/0.717	0.871/0.921/0.795						
EAOU1			0.799	0.886	5.629	5.410	0.861	0.928
EAOU2			0.874	0.883	5.259	5.269	1.060	0.967
EAOU3			0.866	0.905	5.490	5.440	0.971	0.905
Attitude (ATTI)	0.855/0.912/0.775	0.921/0.950/0.863						
ATTI1			0.896	0.937	5.343	5.269	1.062	0.935
ATTI2			0.886	0.931	5.203	5.284	1.098	0.955
ATTI3			0.858	0.919	5.601	5.321	1.056	0.939
Intention (INTE)	0.922/0.951/0.866	0.919/0.949/0.861						
INTEN1			0.932	0.926	5.315	5.299	1.195	0.910
INTEN2			0.926	0.916	4.993	5.269	1.335	1.020
INTEN3			0.933	0.942	5.203	5.246	1.166	0.945

#### 4.1.2 Internal consistency reliability

Internal consistency was assessed by Cronbach’s alpha (CA) or Composite Reliability (CR). The traditional standard for evaluating the internal consistency reliability is CA and a high alpha value indicates that all items in the same construct have the same meaning (Cronbach, 1951). As an indicator of internal consistency reliability, the composition reliability is more accurate than CA (Chin, 1998). CA believes that all indicators are equally reliable. Composition reliability focuses on the differences of different indicators, and different indicators have different loading (Henseler et al., 2009). The CA should be greater than 0.8 and the CR should be greater than 0.7 (Nunnally and Bernstein, 1994). In this study, the CA ranges of BL and OL group were (0.804, 0.922) and (0.871, 0.942) respectively and the CR ranges were (0.884, 0.951) and (0.921, 0.963) respectively (see Table 1). The CA and CR were all greater than the recommended values. It showed that the internal consistency of the measurement model is good.

#### 4.1.3 Convergent validity

The convergent validity reflects the degree of convergence of individual items of a construct compared with items measuring different constructs. A common criterion for convergent validity is the average variance extracted (AVE) (Fornell and Larcker, 1981). An AVE value should be greater than 0.5 to have sufficient convergent validity. In this study, the AVE value ranges of BL group and OL group were (0.717, 0.879) and (0.795, 0.917) respectively (see Table 1), which were larger than the recommended values.

#### 4.1.4 Discriminant validity

Discriminant validity indicates the difference of measurement values of different constructs. It is to check whether the measurement items of one construct have inadvertently measured other constructs. The discriminant validity can be evaluated by two criteria. For the first measure, the square root of the AVE of each latent variable should exceed the correlation between this variable and all other latent variables (Fornell and Larcker, 1981). The second measure, the cross-loadings means that the loading of each indicator of this construct is higher than that of the other construct (Chin, 1998). In the measurement of BL group and OL group, the square root of the AVE of all constructs were greater than that of Pearson correlation coefficient with other constructs (see Table 2). The cross-loadings of BL group and OL group were shown in Table 3. The loading of each indicator on the designated construct was higher than that on other construct (see Table 3). These results show that the constructs of this study have sufficient discriminant validity.

**TABLE 2:** Discriminant validity of the research models of BL and OL.

Construct	ATTI		EAOU		INQU		INSA		INTE		SYQU		SYSA		USEF	
	BL	OL	BL	OL	BL	OL	BL	OL	BL	OL	BL	OL	BL	OL	BL	OL
ATTI	**0.880**	**0.929**														
EAOU	0.758	0.872	**0.847**	**0.892**												
INQU	0.796	0.814	0.728	0.807	**0.918**	**0.938**										
INSA	0.752	0.872	0.686	0.833	0.698	0.858	**0.921**	**0.958**								
INTE	0.807	0.902	0.734	0.897	0.700	0.822	0.660	0.847	**0.930**	**0.928**						
SYQU	0.826	0.863	0.738	0.854	0.828	0.892	0.829	0.903	0.717	0.856	**0.929**	**0.926**				
SYSA	0.871	0.895	0.786	0.861	0.763	0.836	0.823	0.894	0.723	0.865	0.825	0.897	**0.938**	**0.947**		
USEF	0.810	0.884	0.772	0.888	0.756	0.806	0.671	0.849	0.725	0.910	0.734	0.851	0.759	0.874	**0.911**	**0.947**

The bold values in the diagonal row are the square roots of the average variance extracted for the constructs in both research models.

**TABLE 3:** Cross-loadings of the variables in the measurement models of BL and OL.

	ATTI		EAOU		INQU		INSA		INTE		SYQU		SYSA		USEF	
	BL	OL	BL	OL	BL	OL	BL	OL	OL	OL	OL	OL	OL	OL	OL	OL
**Attitude (ATTI)**																
ATTI1	**0.896**	**0.937**	0.663	0.792	0.737	0.748	0.679	0.844	0.690	0.829	0.741	0.801	0.815	0.826	0.728	0.809
ATTI2	**0.886**	**0.931**	0.672	0.825	0.715	0.758	0.705	0.826	0.724	0.842	0.764	0.821	0.778	0.858	0.681	0.844
ATTI3	**0.858**	**0.919**	0.665	0.811	0.650	0.763	0.601	0.760	0.716	0.844	0.676	0.785	0.707	0.812	0.728	0.811
**Ease of use (EAOU)**																
EAOU1	0.528	0.762	**0.799**	**0.886**	0.500	0.747	0.484	0.753	0.548	0.755	0.478	0.772	0.565	0.754	0.573	0.759
EAOU2	0.731	0.803	**0.874**	**0.883**	0.753	0.747	0.715	0.770	0.730	0.869	0.779	0.828	0.767	0.813	0.725	0.832
EAOU3	0.644	0.763	**0.866**	**0.905**	0.567	0.662	0.516	0.701	0.567	0.768	0.581	0.678	0.643	0.732	0.648	0.779
**Information quality (INQU)**																
INQU1	0.737	0.760	0.687	0.772	**0.931**	**0.946**	0.659	0.788	0.660	0.779	0.772	0.843	0.708	0.764	0.703	0.754
INQU2	0.743	0.749	0.657	0.741	**0.924**	**0.937**	0.657	0.786	0.640	0.747	0.718	0.825	0.700	0.787	0.711	0.735
INQU3	0.711	0.780	0.661	0.758	**0.898**	**0.930**	0.604	0.836	0.628	0.786	0.793	0.841	0.693	0.801	0.667	0.775
**Information satisfaction (INSA)**																
INSA1	0.665	0.823	0.627	0.787	0.616	0.805	**0.918**	**0.957**	0.561	0.809	0.731	0.836	0.736	0.859	0.622	0.794
INSA2	0.718	0.846	0.635	0.808	0.669	0.838	**0.923**	**0.959**	0.653	0.812	0.794	0.894	0.778	0.853	0.613	0.831
**Intention (INTE)**																
INTEN1	0.751	0.865	0.712	0.852	0.667	0.781	0.618	0.816	**0.932**	**0.926**	0.655	0.833	0.690	0.861	0.718	0.862
INTEN2	0.727	0.790	0.687	0.807	0.639	0.737	0.603	0.738	**0.926**	**0.916**	0.658	0.754	0.648	0.741	0.638	0.815
INTEN3	0.773	0.854	0.650	0.836	0.648	0.769	0.622	0.800	**0.933**	**0.942**	0.689	0.795	0.680	0.804	0.666	0.855
**System quality (SYQU)**																
SYQU1	0.761	0.792	0.677	0.804	0.791	0.819	0.764	0.830	0.650	0.794	**0.937**	**0.931**	0.777	0.812	0.650	0.779
SYQU2	0.803	0.828	0.724	0.810	0.783	0.818	0.762	0.827	0.700	0.796	**0.942**	**0.917**	0.773	0.841	0.704	0.8
SYQU3	0.735	0.778	0.655	0.758	0.732	0.839	0.785	0.851	0.649	0.789	**0.907**	**0.929**	0.749	0.838	0.691	0.784
**System satisfaction (SYSA)**																
SYSAT1	0.812	0.839	0.718	0.798	0.714	0.808	0.783	0.875	0.684	0.811	0.791	0.849	**0.938**	**0.948**	0.687	0.821
SYSAT2	0.821	0.858	0.755	0.834	0.717	0.776	0.759	0.818	0.672	0.829	0.756	0.851	**0.937**	**0.947**	0.737	0.835
**Usefulness (USEF)**																
USEF1	0.730	0.818	0.672	0.828	0.629	0.766	0.622	0.781	0.650	0.861	0.651	0.813	0.713	0.838	**0.906**	**0.937**
USEF2	0.755	0.855	0.715	0.848	0.716	0.760	0.578	0.817	0.654	0.848	0.669	0.802	0.663	0.816	**0.899**	**0.944**
USEF3	0.729	0.838	0.723	0.845	0.722	0.761	0.634	0.812	0.678	0.876	0.685	0.802	0.700	0.827	**0.929**	**0.958**

The bold values are the loadings of each item on its latent variable in both research models.

### 4.2 Structural model

**FIGURE 1:**
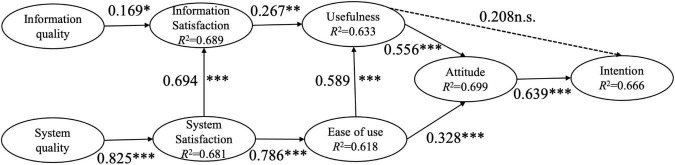
Structural model for the group of blended learning. *p < 0.05; ^**^p < 0.01; ^***^p < 0.001; n.s. = non-significant.

**FIGURE 2:**
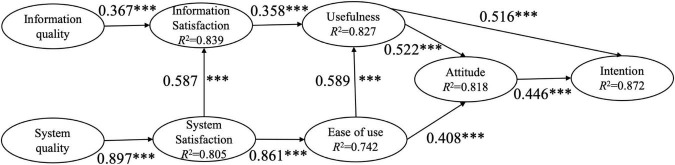
Structural model for the group of online learning. ^***^p < 0.001.

Structural models are mainly evaluated by checking the significance level of the path coefficient in the research model and the explanatory power (i.e., *R*^2^). The validation results of the structural models for the two research designs were presented in Figures 1, 2.

In the structural model of the BL group, information quality and system quality were found to have a significant influence on information satisfaction and system satisfaction, respectively, therefore supporting H1 and H2. Perceived ease of use had a significant positive influence on perceived usefulness, therefore supporting H3. User attitude was significantly affected by perceived ease of use and perceived usefulness, therefore supporting H4 and H6. User attitude was found to have a significant positively influence on usage intention, therefore supporting H7. However, Perceived usefulness was not found to significantly affect usage intention, thus rejecting H5. Information satisfaction had a significant positive influence on perceived usefulness, and system satisfaction had a significant positive influence on perceived ease of use, therefore supporting H8 and H9. System satisfaction was found to have a significant positively influence on information satisfaction, therefore supporting H10. The path coefficient of the BL group model was shown in Figure 1. Meanwhile, in the OL group, all path coefficients were significant, therefore supporting all the ten hypotheses (see Figure 2).

The coefficient of determination (*R*^2^) is the ratio of the interpretable variance of the endogenous latent variables to the total variance in the model, and it is one of the indicators to evaluate the prediction effect of the model. Chin (1998) suggested that when the *R*^2^ of endogenous latent variable is 0.670, it means that the prediction effect of the latent variable is “large”; when the *R*^2^ of the endogenous latent variable is 0.333, the prediction effect of the latent variable is “medium”; when the *R*^2^ of the endogenous latent variable is 0.190, the prediction effect of the latent variable is “small.” In this study, in BL group, the *R*^2^ for information satisfaction, system satisfaction, usefulness, ease of use, attitude and intention were 0.689, 0.681, 0.633, 0.618, 0.699, and 0.666, respectively (see Figure 1). In OL group, *R*^2^ for information satisfaction, system satisfaction, usefulness, ease of use, attitude and intention were 0.839, 0.805, 0.827, 0.742, 0.818, and 0.872, respectively (see Figure 2). Thus, the research models had the predictive power of “medium” or above.

Although PLS-SEM analysis does not have any overall model fit indicators (Henseler et al., 2009). However, Tenenhaus et al. (2004) proposed a global goodness of fit (0 < GoF < 1) criterion for PLS-SEM analysis. It is calculated as the geometric mean of the average communality and average *R*^2^ (Tenenhaus et al., 2004). Wetzels et al. (2009) proposed that the value of GoF is defined as small (0.10), medium (0.25), and large (0.36). The GoF values of BL group and OL group were 0.741 and 0.843, respectively. Therefore, the research models designed in this study had a high degree of fitting. This showed that the two models built in this study were acceptable.

### 4.3 Comparison of path coefficients between blended learning group and online learning group

The previous analysis confirmed the reliability and validity of the two groups of research models. In order to find out whether there is any difference in the research model before and during the COVID-19, this study conducted a multi-group comparative analysis. Generally, the method of comparing path models usually focuses on the differences in the structure level, especially the path coefficient (Sanchez, 2013). In this study, bootstrapping *T*-test was used to compare multiple groups (*K* = 5000). As shown in Table 4, there are differences between BL group and OL group in the paths of “information quality affects information satisfaction” (H1) and “perceived usefulness affects usage intention” (H5).

**TABLE 4:** Comparisons between BL and OL through bootstrapping.

Hypothesis	Path	Group: BL path coefficients	Group: OL path coefficients	diff. abs	*t*	df	*p*	Sig. 05
**H1**	**INQU - > INSA**	**0.169**	**0.367**	**0.198**	**1.985**	**276**	**0.049**	**Yes**
H2	SYQU - > SYSA	0.825	0.897	0.072	1.565	276	0.120	No
H3	EAOU - > USEF	0.589	0.589	0.000	0.004	276	0.997	No
H4	USEF - > ATTI	0.556	0.522	0.034	0.247	276	0.805	No
**H5**	**USEF - > INTE**	**0.208**	**0.516**	**0.308**	**2.096**	276	**0.038**	**Yes**
H6	EAOU - > ATTI	0.328	0.408	0.080	0.556	276	0.579	No
H7	ATTI - > INTE	0.639	0.446	0.193	1.236	276	0.219	No
H8	INSA - > USEF	0.267	0.358	0.091	0.646	276	0.520	No
H9	SYSA - > EAOU	0.786	0.861	0.075	1.441	276	0.152	No
H10	SYSA - > INSA	0.694	0.587	0.107	1.052	276	0.295	No

Diff. abs, absolute difference; df, degree of freedom. The bold values are the values of two hypotheses with significant difference.

### 4.4 Satisfaction and acceptance comparison in two groups

Finally, this study compared the satisfaction and acceptance of college students on the online learning platform between the BL group and the OL group. The *T*-test analysis of the two groups from the three dimensions of information satisfaction, system satisfaction and usage intention showed that there was no difference in the satisfaction and acceptance of online learning platform between the two groups, as shown in Table 5.

**TABLE 5:** The two groups were analyzed by *T*-test in intention, information satisfaction and system satisfaction.

Construct	Group	N	M	SD	*T*-test
INTEN	BL	143	5.170	1.146	*t* = −0.822, *p* = 0.412
	OL	134	5.271	0.889	
INSA	BL	143	5.455	0.925	*t* = 1.170, *p* = 0.243
	OL	134	5.325	0.922	
SYSA	BL	143	5.350	0.971	*t* = 0.291, *p* = 0.771
	OL	134	5.317	0.877	

## 5 Discussion

The purpose of this study was to investigate and analyze the influence factors of learners’ satisfaction and acceptance of online learning platform based on USATA model, and focused on the comparative investigation and analysis of the differences in the context of online learning and blended learning. The results of data analysis showed that there was no significant difference in college students’ satisfaction and acceptance in both groups, but the information quality of online learning platform had a greater impact on college students’ information satisfaction during the home-based online learning, and there was a significant difference between the two groups. In blended learning environment, the perceived usefulness of online learning platform had no significant impact on college students’ intention to use the online learning platform. In online learning environment, the perceived usefulness of online learning platform significantly affected college students’ intention to use the online learning platform, and the degree of impact was large.

### 5.1 Information quality and system quality affect user’s online learning platform satisfaction

From the research results, we can see that in the level of students’ satisfaction with online learning platforms, the proposed hypotheses 1, 2, and 10 have been supported, and the information quality has a significant positive impact on information satisfaction; System quality has a significant positive impact on system satisfaction; System satisfaction has a significant positive impact on information satisfaction. In online learning environment, the quality of learning resources provided by online learning platform will affect college students’ satisfaction with online learning content. The quality of online learning platform itself will affect college students’ satisfaction with online learning platform. These results have been supported in the research of information system satisfaction measurement, and are consistent with the existing research results (Doll and Torkzadeh, 1988). Online learning platform is also a specific form of information system. This study focused on whether students’ satisfaction with online learning platforms was different in different learning environments.

Through investigation and analysis, it was found that there was a significant difference in the path of information quality affecting information satisfaction between blended learning and online learning environments. In online learning environment, information quality had a greater impact on user’s information satisfaction. To some extent, this showed that college students had higher requirements for the quality of learning resources provided by the online learning platform during the home-based online learning. The quality of online learning resources was directly related to learners’ satisfaction with online learning. The previous studies on online learning and online learning platforms during the COVID-19 also showed that the learning method had changed from face-to-face learning to full online learning. The interaction between teachers and students and the acquisition of online learning resources in the online learning environment were particularly important (Almusharraf and Khahro, 2020; Tsang et al., 2021). The results of this study can be explained from two aspects. On the one hand, in face-to-face learning environment, online learning platform is not a necessary way for students to obtain information. It is more convenient for students to communicate with teachers and peers. Learning resources can be obtained through teacher-student conversation, or through libraries and other channels. However, during the period of full online learning at home, learning mainly relies on an online learning platform, and students will naturally obtain learning resources from the online learning platform. Therefore, college students have higher requirements for the quality of learning resources provided by the online platform. On the other hand, the data analysis results of this study verified the impact of system satisfaction on information satisfaction. This showed that personal satisfaction with the system may affect their satisfaction with the system information. Therefore, learners’ satisfaction with the online learning platform will affect learners’ satisfaction with the learning resources provided by the online learning platform. This result may be because college students are more satisfied with the online learning platform they use, and will be more accustomed to using the online learning platform to learn and obtain learning resources and information, so they will be more satisfied with the learning information provided in the platform.

### 5.2 Perceived usefulness and perceived ease of use affect user attitude and usage intention

The data analysis results of this study showed that in the level of students’ acceptance of online learning platforms, the proposed hypotheses 3–7 have been supported in the OL group, while in BL group, except H5, H3, H4, H6, and H7 were also supported. Perceived usefulness and perceived ease of use will affect user attitudes toward online learning platforms, which will affect their intention to use. Perceived ease of use affects perceived usefulness. Students perceive that the online learning platform is useful or easy to use. In this way, students like to use the online learning platform and have a more positive attitude toward the online teaching platform, resulting in a stronger intention to use the online learning platform. Students perceive that online learning platforms are easy to use, which will also lead to the perception that online learning platforms are more useful. These results are consistent with those of the TAM (Davis et al., 1989; Davis and Wiedenbeck, 2001). The results of this study were also consistent with previous studies results of investigating the acceptance of other online learning platforms with the TAM model (Sánchez and Hueros, 2010; Zhou et al., 2022).

Through the comparative analysis of the models of blended learning and complete online learning, it was found that there was no significant difference between the two groups in the impact of perceived usefulness and perceived ease of use on user attitude, and there was no significant difference in the impact of perceived ease of use on perceived usefulness, but there was a significant difference in the impact of perceived usefulness on usage intention. In blended learning environment, the hypothesis that perceived usefulness affects learners’ intention to use online learning platforms was not supported, which showed that user perception of the usefulness of online learning resources did not significantly affect learners’ intention to use them. This is different from some previous studies. This difference may be caused by the fact that students mainly studied by face-to-face way in the classroom environment, and learners had many ways to obtain learning resources, so their dependence on online learning platforms was not obvious, that is, even if the learning resources provided by online learning platforms were very useful, students did not necessarily use them. In complete online learning environment, learners’ perceived usefulness will significantly affect their intention to use online learning platforms, and the degree of influence is greater. This can explain that in the complete home-based online learning environment, online learning platform has become the important way for students to obtain learning resources. Learners pay more attention to the usefulness and quality of online learning resources. If the online learning platform provides high-quality and useful learning resources, learners are more willing to use the online learning platform. This result was consistent with the conclusion in the satisfaction survey that “the quality of learning resources affects learners’ online learning satisfaction.”

### 5.3 Online platform satisfaction affects the perception of usefulness and ease of use behavior beliefs, and then affects the usage intention

In this study, hypotheses 8 and 9 were supported in the relationship between college students’ satisfaction and acceptance of online learning platforms. Research showed that information satisfaction affects user perceived usefulness and system satisfaction affects user perceived ease of use. If learners are satisfied with online learning resources, they think that learning resources are more useful; If learners are satisfied with the online learning platform, they will think that the platform is easy to use. These results were consistent with those proposed and verified by USATA model (Wixom and Todd, 2005). In the blended learning group, the impact of information satisfaction on perceived usefulness was slightly smaller than that in the online learning group, but there was no significant difference between the two groups.

In addition, the study found that there was no significant difference in learners’ satisfaction and acceptance of the online learning platform in two groups. The reason for this result may be related to the fact that the participants of this study are college students who usually have more contact with online learning platforms. These students have been using the online learning platform for blending learning in their professional learning, and are familiar with the online learning platform. Another reason may be related to the online learning platform investigated. The online learning platform investigated in this study was Chaoxing Fanya platform. The university where the respondents are located has been using this platform to carry out public network digital courses, and many professional courses also use this platform for auxiliary teaching. It can be seen that students are more accustomed to using the online learning platform at ordinary times, so it will be more acceptable to continue to use the online learning platform for online learning at home, and they will be more satisfied with the online learning platform and the learning resources it provides.

## 6 Conclusion and limitation

This study focused on college students’ satisfaction and acceptance with the online learning platform and its key influence factors, and verified the USATA model in two learning environments. The study found that during the home-based online learning, the quality of learning resources provided by online learning platforms has a more significant impact on students’ satisfaction and acceptance of online learning platforms. This study integrated learners’ satisfaction and acceptance into a model to investigate learners’ intention to use online learning platforms in different learning environments, which provides a new perspective for similar studies. Moreover, the results of this study also provided a basis for the design and development of online learning platform, that is, we should pay attention to the external factors that affect user’s satisfaction and acceptance, and start from improving the quality of online learning platform itself and online learning information. Only by focusing on the quality of online learning platform and resources, and ensuring the ease of use of the platform and the usefulness of resources, can learners’ satisfaction and acceptance of online learning be improved. In order to improve learners’ satisfaction and acceptance of online learning platform, online learning platform designers and developers should focus on the quality of online learning platform itself and the information it provides, so as to improve the ease of use of online learning platform and the usefulness of learning resources.

The results of this study have two important implications for the practical activities of development and application of online learning platform. The first implication is that the quality of online learning platform affects learners’ satisfaction and usage intention, so the design and development of online learning platform should focus on its quality. When developing online learning platform, developers can improve the quality of online learning platform from the aspects of reliability, accessibility, flexibility, comprehensiveness, timeliness and so on (Ives et al., 1983). First of all, we should ensure that the online learning platform is stable and reliable, without congestion and collapse, and convenient for learners to use. The second is to ensure that learners can easily obtain the required resources and tools through the platform. Third, enrich the functions of the online platform to meet different learning needs. Fourth, integrate the learning functions commonly used in online learning, collect different types of learning resources, and be compatible with the resources and tools of other learning platforms. Fifth, give timely feedback to learners’ interaction to ensure smooth human-computer interaction.

The second implication is that the quality of online learning resources affects learners’ satisfaction and intention to use resources. Therefore, the design and development of online learning resources should ensure their quality. Online learning resources can ensure the quality of resources from their integrity, standardization, accuracy, currency and ease of use (Bailey and Pearson, 1983). First, online learning resources should provide complete and rich resources for learners’ needs. Second, online learning resources should be standardized in presentation style and clear in content design. Third, the accuracy of online learning resources should be guaranteed, and there should be no wrong content. Fourth, the content of online learning resources should be constantly updated to provide the latest learning content. Fifth, online learning resources need to be carefully designed, such as learning units, learning paths, learning activities, learning content presentation and so on, which need to be reasonably designed to ensure that learners can easily access and use.

This study has two limitations that should be addressed in future research. First, the online learning platform surveyed is relatively single. The online learning platform investigated in this study is Chaoxing Fanya platform, which cannot explain the quality of all online learning platforms. This research limitation is also reflected in the research results. The results of this study showed that there was no significant difference in students’ satisfaction and acceptance of Chaoxing Fanya platform in two groups. This may be related to the fact that the respondent groups are familiar with the learning platform. The second limitation is that the level of respondents is not diverse enough to involve primary and secondary school learners. For primary and secondary school students who had less access to online learning platforms in face-to-face teaching at schools, their satisfaction and acceptance of online learning platforms may be more different. Although this study has some limitations, it also provides the research direction in related research fields to a certain extent. According to the idea of this study, it is necessary to investigate the USATA model of more other countries during the COVID-19 and after the normalization of the epidemic, analyze the factors that affect the satisfaction and acceptance of online learning platforms, and provide educational support to students in a wider range.

## Data availability statement

The original contributions presented in this study are included in the article/supplementary material, further inquiries can be directed to the corresponding author.

## Author contributions

XL conducted online teaching, found research problems, designed research plans, and conducted investigation research. WZ processed and analyzed the research data. Both authors participated in the writing, revision and submission of the manuscript.

## Appendix

**APPENDIX TABLE A1:** Dimensions and items of the research model.

Construct	Item
Information quality (INQU)	INQU 1. In a word, I highly evaluate the content on learning platform
	INQU 2. In short, I highly value the functions of the learning platform
	INQU 3. In short, the resources on the learning platform are of high quality.
System quality (SYQU)	SYQU 1. Regarding the product quality of learning platform, I give a very high evaluation.
	SYQU 2. In short, learning platform has high quality in terms of function.
	SYQU 3. In short, the resources provided by learning platform are of high quality.
Information satisfaction (INSA)	INSA 1. I am satisfied with the learning resources on learning platform.
	INSA 2. I am very satisfied with the learning resources provided by learning platform.
System satisfaction (SYSA)	SYSA 1. Considering all aspects, I am very satisfied with the learning platform.
	SYSA 2. I am very satisfied with the interactive experience with the learning platform.
Ease of use (EAOU)	EAOU 1. The learning platform is easy to use.
	EAOU 2. On the learning platform, it is easy to operate and realize the functions I want.
	EAOU 3. It is very easy to operate on the learning platform.
Usefulness (USEF)	USEF 1. Using the learning platform is helpful to improve my learning methods.
	USEF 2. Learning platform saves time and improves efficiency.
	USEF 3. Learning platform has improved my learning quality.
Attitude (ATTI)	ATTI1. I like the process of using learning platform very much.
	ATTI 2. In short, the experience of using learning platform is happy.
	ATTI 3. I am in favor of using learning platform.
Intention (INTE)	INTE 1. In the future, I plan to use more learning platform in my learning process.
	INTE 2. As long as I have the opportunity to use learning platform, I will seize every opportunity to use it.
	INTE 3. I plan to increase the frequency of using learning platform in the future.

The above scales were used for online learning and blended learning, respectively. The formulation of online learning and blended learning scenarios had been omitted in the table.
